# Flight of the PEGASUS? Comparing Transformers on Few-Shot and Zero-Shot Multi-document Abstractive Summarization

**Published:** 2020-12

**Authors:** Travis R. Goodwin, Max E. Savery, Dina Demner-Fushman

**Affiliations:** U.S. National Library of Medicine, National Institutes of Health

## Abstract

Recent work has shown that pre-trained Transformers obtain remarkable performance on many natural language processing tasks, including automatic summarization. However, most work has focused on (relatively) data-rich single-document summarization settings. In this paper, we explore highly-abstractive multi-document summarization, where the summary is explicitly conditioned on a user-given topic statement or question. We compare the summarization quality produced by three state-of-the-art transformer-based models: BART, T5, and PEGASUS. We report the performance on four challenging summarization datasets: three from the general domain and one from consumer health in both zero-shot and few-shot learning settings. While prior work has shown significant differences in performance for these models on standard summarization tasks, our results indicate that with as few as 10 labeled examples, there is no statistically significant difference in summary quality, suggesting the need for more abstractive benchmark collections when determining state-of-the-art.

## 1 Introduction

Since its inception (Luhn, 1958), automatic summarization has focused on summarizing documents either in a generic way – conveying the main points of the document to any reader regardless of their information need – or in a task-specific way – distilling the important points of the document with respect to a specific information need such as a question or topic statement (Mani, 2009). In the latter case, the selection of the most salient points in the document (i.e., content selection) as well as the expression of those points (i.e., surface realization) must be explicitly conditioned on a user-given natural language context statement, such as a question or topic of interest. In this setting, a single passage may be summarized in different ways depending on the context description. Consequently, obtaining reference summaries is often time- or cost-prohibitive, particularly when dealing with specialized domains such as healthcare.

**Figure 1:**
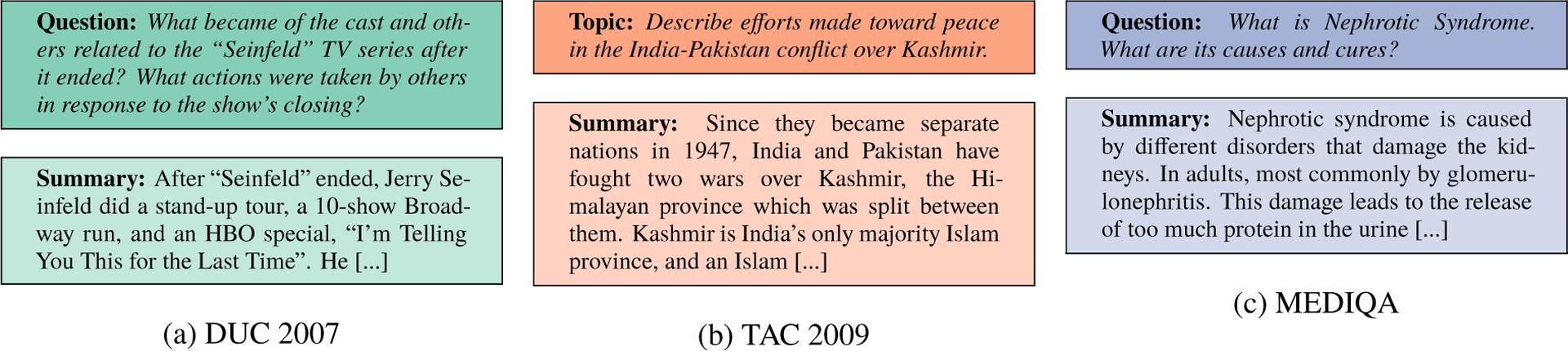
Example topic- and question-driven multi-document abstractive summaries (documents omitted).

The Document Understanding Conference has explored Topic-driven summarization (DUC) and its successor, the Text Analysis Conference (TAC), which both ran community evaluations of topic- or question-based summarization. Specifically, participants were asked to develop automatic summarization approaches for generating single- or multi-document summaries that summarized a set of documents with respect to a given topic description or question, as shown in Figure 1. Human assessors manually judged submitted summaries.

In this work, we revisit the multi-document topic-driven abstractive summarization datasets produced from DUC 2007, TAC 2009, and TAC 2010, as well as question-driven summarization from consumer health. Because these datasets are relatively small (approximately 45 topics each), we explore modern transformer-based models’ performance in the zero-shot and few-shot (10 examples) learning settings. Specifically, we explore the quality of multi-document abstractive summarization generated by T5 (Raffel et al., 2019), BART (Lewis et al., 2019), and PEGASUS (Zhang et al., 2019).

## 2 Background

Recent work has indicated that transfer learning (pre-training a model on data-rich tasks before fine-tuning it on a downstream task) obtains remarkable performance on many natural language processing tasks (Yang et al., 2019; Dong et al., 2019; Liu et al., 2019b). The most successful models are obtained through self-supervised pre-training with massive datasets to obtain transferable knowledge for new tasks (i.e., fine-tuning) with less abundant data (Devlin et al., 2019; Lewis et al., 2019; Keskar et al., 2019; Raffel et al., 2019). More recently, research has indicated that these models can generate language conditioned on a user-given prompt or context. For example, this prompt can guide the model’s content selection towards a particular topic (Keskar et al., 2019) or inform surface realization for a specific task (Lewis et al., 2019; Raffel et al., 2019). In Liu et al. (2020), the authors condition an extractive transformer using “control codes” to specify the position, importance, and diversity of the sentences in the source text. In this work, we adapt this paradigm to train and evaluate BART, T5, and PEGASUS for abstractive multi-document summarization.

Although zero-shot learning (ZSL) has received considerable attention in the image processing community, there has been comparatively little work on zero-shot learning specifically for summarization: Duan et al. (2019) explore zero-shot learning for cross-lingual sentence summarization and Liu et al. (2019a) explored zero-shot abstractive summaries of five-sentence stories. We extend these works by evaluating zero-shot and few-shot learning for multi-document abstractive summarization.

## 3 Models

In this work, we compare three of the most prominent conditional language generation models: T5, BART, and PEGASUS. To facilitate comparison, for each model we chose the variant with the most similar architecture (such that each consists of 12 transformer layers and a similar number of learnable parameters). Each model is pre-trained with unique strategies as described below.

### BART

BART (Bidirectional and Auto-Regressive Transformers) is pre-trained on document rotation, sentence permutation, text-infilling, and token masking and deletion objectives (Lewis et al., 2019). In our experiments, we used BART-Large.

### T5

T5 (Text-to-Text Transfer Transformer) is pre-trained on several unsupervised and supervised objectives, such as token and span masking, as well as translation, classification, reading comprehension, and summarization. Importantly, each objective is treated as a language-generation task, where the model is conditioned to generate the correct output based on a textual prompt included in the input sequence (Raffel et al., 2019). In this work, we used T5-Base.

### PEGASUS

PEGASUS (Pre-training with Extracted Gap-sentences for Abstractive SUmmarization Sequence-to-sequence) was specifically designed for abstractive summarization and is pre-trained with a self-supervised gap-sentence-generation objective (Zhang et al., 2019). In this task, entire sentences are masked from the source document, concatenated, and used as the target “summary”. We used PEGASUS-Base in our experiments.

## 4 Experiments

We evaluated T5, BART, and PEGASUS in zero-shot (ZSL) and few-shot (FSL) learning settings on four datasets. Summary quality was measured using ROUGE-1, ROUGE-2, and ROUGE-L *F*_1_-scores (Lin, 2004); BLEU-4 (Papineni et al., 2002); and Repetition Rate (in unigrams). Implementation details are provided in Appendix A.

### 4.1 Answer Summarization at DUC 2007

The 2007 challenge of the Document Understanding Conference (DUC) focused on answering 45 natural language questions by summarizing sets of 10 documents from the AQUAINT English news corpus (Graff, 2002). Reference summaries were between 230 and 250 words. We used 30 topics for testing (with 10 for training and 5 for validation under FSL). Table 1 presents these results, showing that BART obtains the highest quality summaries in both settings, though FSL provides a significant increase for all models.

### 4.2 Update Summarization at TAC 2009

In 2009, the Text Analysis Conference (TAC) summarization evaluation explored summarizing sets of 10 newswire articles with respect to a given topic description in approximately 100 words under the assumption that a user had already read a given set of earlier articles (Dang and Owczarzak, 2009). Of 44 topics used in 2009, we used 30 for testing (with 10 for training and 4 for validation under FSL). Table 2 presents these results. While T5 had the highest performance in zero-shot performance, there was no statistically significant difference in terms of ROUGE after few-shot training, although T5 did obtain improved BLEU.

### 4.3 Guided Summarization at TAC 2010

Similar to the 2009 evaluation, the summarization track’s goal in TAC 2010 was to produce 100-word summaries of sets of 10 newswires articles for 46 given topics. However, in 2010 each topic was assigned to one of five pre-defined categories, and summaries were expected to cover all aspects associated with that category (e.g., for *Accidents and Natural Disasters*, summaries should cover (a) what happened, (b) when it happened, (c) the reasons for the accident or disaster, (d) casualties, (e) damages, and (f) rescue efforts or countermeasures) (Owczarzak and Dang, 2010). We used 30 topics for testing (with 10 for training and 6 for validation). Results are illustrated in Table 3. In this case, BART had the highest performance in both ZSL and FSL settings, although FSL provided significant improvements for all models, allowing T5 to obtain similar ROUGE-2 and ROUGE-L performance.

### 4.4 MEDIQA Summarization

The MEDIQA collection contains consumer health questions, sets of passages extracted from reliable websites relevant to the question, and human-authored multi-document summaries of the passages intended to provide consumer-friendly answers (Savery et al., 2020). Of the 156 available abstractive multi-document summaries, we used 141 questions for testing (with 10 for training and 5 for validation under FSL). Table 4 provides these results. While FSL provided a clear improvement for all models, there were no statistically significant differences in summary quality between the three models using FSL. Example summaries from all systems for a single MEDIQA question are provided in Figure 3.

### 4.5 Few-shot and Zero-shot Learning

**Figure 2:**
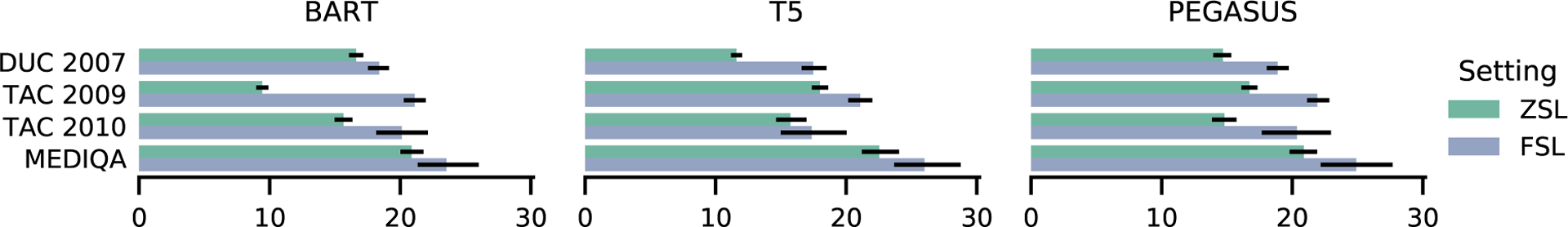
Rouge-L of each model trained under the few-shot (FSL) and zero-shot (ZSL) learning settings.

**Figure 3:**
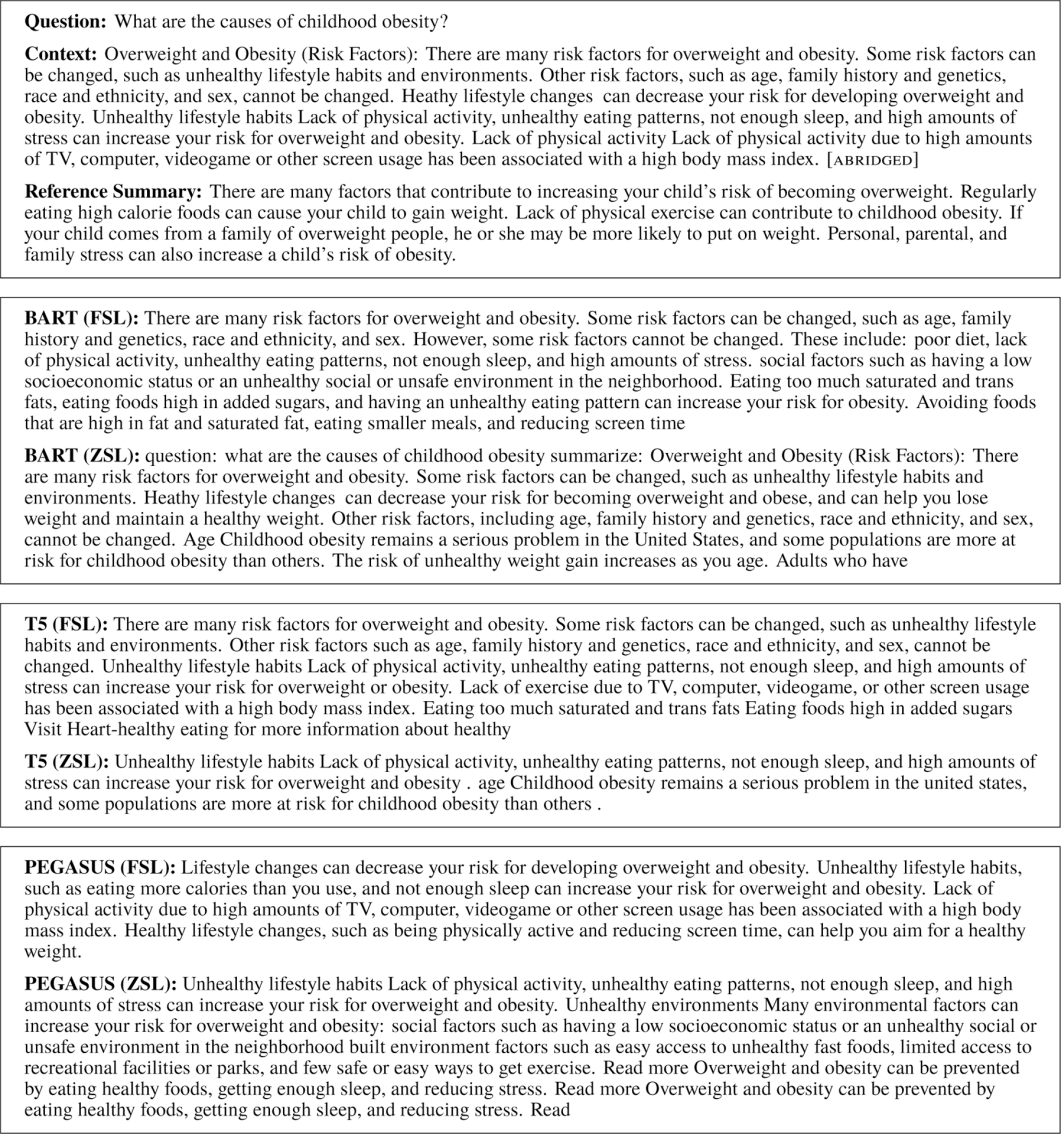
Example summaries for the question, *What are the causes of childhood obesity?*

Figure 2 compares the performance of each model in FSL and ZSL settings. FSL provided significant increases in performance on all tasks for PEGASUS, all but MEDIQA for BART, and only two tasks for T5, suggesting that while FSL is clearly useful for all three models, it most benefits PEGASUS.

## 5 Conclusion

We evaluated the summarization quality produced by three state-of-the-art transformers: BART, T5, and PEGASUS on four challenging summarization dataset in both zero-shot and few-shot learning settings. Our results indicate that, while there are statistically significant differences between the models in zero-shot settings, after few-shot learning with as few as 10 examples, there is little discernible difference between them. This suggests that while large improvements have been made on standard single-document benchmarks, highly abstractive multi-document summarization remains challenging.

## Figures and Tables

**Table 1:** Abstract multi-document summarization on DUC 2007 with 95% confidence intervals.

System	ROUGE-1	ROUGE-2	ROUGE-L	BLEU-4	Repetition
T5 (ZSL)	21.21 (20.37 – 22.04)	4.35 (3.82 – 4.91)	11.59 (11.17 – 12.03)	1.45 (1.24 – 1.73)	33.21 (31.82 – 34.60)
T5 (FSL)	36.35 (34.96 – 37.66)	9.12 (8.27 – 9.94)	17.46 (16.85 – 18.10)	4.81 (4.22 – 5.51)	54.20 (52.27 – 56.24)
BART (ZSL)	37.36 (36.18 – 38.59)	8.08 (7.34 – 8.88)	16.62 (16.08 – 17.18)	5.14 (4.52 – 5.84)	44.91 (44.05 – 45.83)
BART (FSL)	**40.86** (39.84 – 41.81)	**9.40** (8.69 – 10.08)	18.38 (17.93 – 18.85)	**6.06** (5.46 – 6.68)	53.96 (53.17 – 54.69)
PEGASUS (ZSL)	26.36 (25.05 – 27.64)	5.01 (4.38 – 5.70)	14.69 (13.95 – 15.34)	2.18 (1.83 – 2.58)	65.52 (60.81 −70.36)
PEGASUS (FSL)	36.02 (34.63 – 37.33)	7.95 (7.26 – 8.65)	**18.88** (18.27 – 19.49)	5.21 (4.57 – 5.85)	74.29 (71.73 – 76.92)

**Table 2:** Abstract multi-document summarization on TAC 2009 with 95% confidence intervals.

System	ROUGE-1	ROUGE-2	ROUGE-L	BLEU-4	Repetition
T5 (ZSL)	29.97 (28.55 – 31.38)	9.03 (7.78 – 10.30)	17.98 (16.89 – 19.21)	3.67 (3.10 – 4.36)	27.96 (26.76 – 29.31)
T5 (FSL)	38.36 (36.92 – 39.85)	**11.56** (10.25 – 12.95)	21.06 (19.74 – 22.59)	**8.55** (7.32 – 9.78)	33.91 (32.75 – 35.03)
BART (ZSL)	12.82 (11.68 – 13.97)	3.73 (3.27 – 4.20)	9.43 (8.76 – 10.12)	0.57 (0.44 – 0.74)	7.32 (5.43 – 9.41)
BART (FSL)	**39.28** (38.10 – 40.46)	11.33 (10.37 – 12.44)	21.11 (20.27 – 22.02)	7.30 (6.49 – 8.11)	45.30 (44.24 – 46.33)
PEGASUS (ZSL)	25.69 (23.88 – 27.66)	5.70 (4.74 – 6.69)	16.72 (15.77 – 17.65)	3.31 (2.81 – 3.91)	75.56 (71.07 – 80.36)
PEGASUS (FSL)	38.96 (37.64 – 40.17)	10.44 (9.51 – 11.40)	**21.92** (20.84 – 22.95)	7.00 (6.24 – 7.88)	43.59 (41.50 – 45.87)

**Table 3:** Abstract multi-document summarization on DUC 2007 with 95% confidence intervals.

System	ROUGE-1	ROUGE-2	ROUGE-L	BLEU-4	Repetition
T5 (ZSL)	27.01 (25.65 – 28.35)	6.25 (5.35 – 7.29)	15.72 (14.84 – 16.75)	2.06 (1.72 – 2.45)	30.47 (29.07 – 31.91)
T5 (FSL)	34.13 (32.72 – 35.77)	8.36 (7.32 – 9.50)	17.35 (16.44 – 18.28)	5.59 (4.70 – 6.54)	32.60 (31.25 – 34.05)
BART (ZSL)	28.97 (27.48–30.70)	6.32 (5.58 – 7.24)	15.64 (14.80 – 16.40)	3.62 (3.11 – 4.22)	27.96 (26.06 – 29.74)
BART (FSL)	**38.22** (36.98 – 39.41)	**10.15** (9.17 – 11.18)	20.11 (19.27 – 20.94)	**6.85** (5.99 – 7.68)	39.91 (38.67 – 41.11)
PEGASUS (ZSL)	24.87 (23.14–26.48)	4.99 (4.31 – 5.77)	14.80 (13.97 – 15.65)	2.66 (2.26 – 3.19)	57.15 (51.71 – 62.81)
PEGASUS (FSL)	36.31 (34.95 – 37.63)	9.21 (8.27 – 10.15)	**20.35** (19.56 – 21.26)	5.81 (5.09 – 6.62)	40.39 (37.73 – 43.31)

**Table 4:** Abstract multi-document summarization on MEDIQA with 95% confidence intervals.

System	ROUGE-1	ROUGE-2	ROUGE-L	BLEU-4	Repetition
T5 (ZSL)	31.09 (28.46 – 33.72)	14.63 (11.77 – 17.58)	22.52 (20.15 – 25.19)	7.12 (5.07 – 9.36)	31.00 (29.06 – 32.96)
T5 (FSL)	**38.56** (35.94 – 41.13)	**18.52** (15.54 – 21.50)	**26.00** (23.67 – 28.76)	10.90 (9.08 – 13.07)	36.19 (34.73 – 37.78)
BART (ZSL)	33.51 (31.21 – 36.14)	13.87 (11.52 – 16.31)	20.87 (18.92 – 22.88)	8.21 (6.38 – 10.18)	38.24 (36.60 – 39.79)
BART (FSL)	37.65 (35.07 – 40.37)	17.01 (14.38 – 20.12)	23.54 (21.34 – 26.00)	10.83 (8.83 – 13.04)	41.48 (40.13 – 42.87)
PEGASUS (ZSL)	29.75 (26.20 – 32.89)	12.17 (9.44 – 15.12)	20.88 (18.19 – 23.49)	8.61 (6.53 – 10.84)	63.87 (58.64 – 69.69)
PEGASUS (FSL)	37.02 (33.86 – 40.33)	17.04 (13.95 – 20.12)	24.90 (22.18 – 27.68)	**12.40** (9.96 – 15.08)	46.81 (43.40 – 50.27)

## Appendix A Implementation Details

Metrics, confidence intervals, and the PEGASUS implementation were provided by https://github.com/google-research/pegasus. All models were trained with a batch size of 8, maximum sequence length of 512 tokens, and 3 warm-up epochs followed by 20 training epochs using single V100X GPUs (32 GB VRAM) on a shared cluster. Validation loss was measured every epoch and the snapshot with lowest validation loss was used for FSL evaluation. Documents were sorted by similarity to their reference summaries when provided to the model. T5 and BART implementations were provided by HuggingFace’s Transformers package (Wolf et al., 2019). Existing datasets were obtained using the TensorFlow DataSets catalogue. The source code for this paper is available on GitHub at https://github.com/h4ste/mdas.
